# Exploring trajectories of functional decline and recovery among older adults: a data-driven approach

**DOI:** 10.1038/s41598-024-56606-0

**Published:** 2024-03-15

**Authors:** Ghazal Khalili, Manaf Zargoush, Kai Huang, Somayeh Ghazalbash

**Affiliations:** 1 DeGroote School of Business, McMaster University, Hamilton, ON L8S 4L8 Canada; 2 Smith School of Business, Queen’s University, Kingston, ON K7L 2P3 Canada

**Keywords:** Quality of life, Geriatrics, Disability

## Abstract

Independently performing activities of daily living (ADLs) is vital for maintaining one’s quality of life. Losing this ability can significantly impact an individual’s overall health status, including their mental health and social well-being. Aging is an important factor contributing to the loss of ADL abilities, and our study focuses on investigating the trajectories of functional decline and recovery in older adults. Employing trajectory analytics methodologies, this research delves into the intricate dynamics of ADL pathways, unveiling their complexity, diversity, and inherent characteristics. The study leverages a substantial dataset encompassing ADL assessments of nursing home residents with diverse disability profiles in the United States. The investigation begins by transforming these assessments into sequences of disability combinations, followed by applying various statistical measures, indicators, and visual analytics. Valuable insights are gained into the typical disability states, transitions, and patterns over time. The results also indicate that while predicting the progression of ADL disabilities presents manageable challenges, the duration of these states proves more complicated. Our findings hold significant potential for improving healthcare decision-making by enabling clinicians to anticipate possible patterns, develop targeted and effective interventions that support older patients in preserving their independence, and enhance overall care quality.

## Introduction

Activities of daily living (ADLs) are fundamental tasks required for maintaining independent living and social engagement, directly impacting an individual’s quality of life^1,2^. Different factors may contribute to a decline in the ability to perform ADLs, e.g., age, socioeconomic conditions, and pain^3^. In the United States, 70% of individuals aged 65 or older require ongoing assistance for the rest of their lives due to at least two ADL losses^4^. Also, ADL disabilities are associated with mental health issues; particularly, patients in a severe stage of ADL loss encounter considerable disadvantages in various social well-being indicators, such as fewer friendships^5^. Thus, individuals experiencing ADL decline are prone to feelings of loneliness and a diminished sense of social belonging. Moreover, the ability to perform ADLs is an indicator of functional status, which is why it is regarded as the “sixth vital sign”, representing a primary assessment of an individual’s overall health^6,7^. As the aging population grows, understanding the dynamics of the complex system in aging becomes increasingly relevant^8^. These systemic changes often manifest in daily life activities, making the examination of functional status and ADLs more prevalent and imperative among the elderly^3,9^, particularly in today’s aging world^10^.

Understanding ADL trajectories is vital for decision-makers to intervene better^11^. Data-driven techniques that analyze electronic health records (EHRs) can help empirically identify patterns and changes in these trajectories^12^. Among these techniques, process mining, machine learning, and latent class models have found their place in trajectory analysis. However, they often fall short of capturing the intricate temporal sequencing and event patterns in a patient’s healthcare journey^12^. Sequence analysis (SA) emerges as an innovative approach derived from social sciences to delve into the ordered sequences of healthcare events and classify individuals into groups exhibiting similar patterns^12^. Unlike process mining, which focuses on optimizing patient flow, or latent class analysis that categorizes patients based on shared characteristics, SA provides unique insights into the temporal dynamics and sequential patterns in patient care^12^. SA’s potential in healthcare research is evident, offering insights into treatment patterns and their effectiveness, which can inform the redesign and optimization of care pathways^12^. Nevertheless, the application of SA in the context of ADL trajectories is relatively unexplored. Expanding research in this area can contribute significantly to a more nuanced understanding of how older individuals encounter activities of daily living over time.

In the field of ADL trajectory analysis, a range of studies have employed various methodologies to understand disability progression. A systematic review examined disability progression in specific chronic illnesses^9^. Several studies explored recovery trends and ADL status pre- and post-hospitalization^13–15^, with assessing the impact of exercise programs on ADL changes^15^. A predictive approach was taken to develop a tool for assessing and forecasting ADLs in nursing home patients^16^. Collectively, these studies enhance the understanding of ADL trajectories, emphasizing the importance of interventions and predictive tools in different healthcare contexts. Despite these efforts, the existing literature mainly examined ADL trajectories from a broad perspective, often relying on ADL scores to investigate the overall trends or levels, with less emphasis on the detailed sequences of functional loss or recovery^14,15,17^. Only a few studies have examined the actual sequences of functional loss and recovery in the context of ADLs^18^. This study is inherently exploratory in nature, aiming to define ADL trajectories as the sequences of disability states that individuals experience. We analyze the trajectories that patients undergo as they either face functional decline or recover from such disabilities using SA. To our knowledge, no prior study has explored trajectories of functional decline and recovery through trajectory analytics (i.e., sequence-based). SA is employed in our study for its capability to analyze diverse aspects of ADL trajectories, providing insights into the ordering, timing, and duration of disability states^19^, thus contributing to a deeper understanding of ADL trajectories.

## Methods

### Data source and study population

The data for our study was sourced from the veterans affairs (VA) Corporate Data Warehouse and processed using the VA Computing Infrastructure^16^. It included data from the VA’s Electronic Medical Record System and nationwide Minimum Data Set (MDS) assessments conducted in VA nursing homes, also known as community living centers (CLCs). MDS, being a uniform and mandatory clinical assessment tool, offers extensive evaluations across various health areas. Collected by specialized staff, this data is sent to a central database for quality control and resource management purposes^18^.

The study sample comprises 1,328,052 records of ADL assessments conducted on 265,530 residents from CLCs, spanning from January 1, 2000, to October 9, 2012. Each resident has undergone a varying number of assessments, which enables the effective tracing of their individual ADL trajectories. The data encompasses assessment results on nine essential physiological functions (including bathing (*B*), grooming (*G*), dressing (*D*), feeding (*F*), transferring (*S*), walking (*W*), toilet using (*T*), bowel continence (*L*), and urinary continence (*U*)), demographic information (including age and sex), unique IDs, initial and final disability dates, follow-up duration, number of completed assessments, and mortality status. For 265,523 residents with valid age values, the average age at the first assessment was 71.07 years (SD = 12.26), with a predominantly male population (96.9%). For detailed information on data sourcing and ADL coding, please refer to the Supplementary online (Appendix A).

### Data preparation

We excluded records of residents with invalid age values, which constituted a minor portion (0.004% or 55 records) of the dataset. Single-assessment cases and death records were also excluded, as death is an irreversible state and inherently distinct from ADL states. After this data preprocessing, 846,859 ADL assessment records remained for analysis. In our study, each physiological function (or simply ADL disability) was assessed at two distinct levels: (0) representing independence or partial dependence, and (1) indicating complete dependence^18^. It is important to note that in this context, “state” refers to specific combinations of these ADL disabilities. For example, *GBW* indicates the presence of grooming, bathing, and walking disabilities. Given our assessment of 9 ADL disabilities, each with two levels, there are theoretically 512 ($2^9$) possible disability combinations (i.e., states), but many are infrequent. To streamline our analysis and maintain computational efficiency, we concentrated on 25 predominant disability states, covering 84.3% of all observed cases. Additional details regarding the dataset, the state extraction process (binary coding the states’ names), and the 25 selected states can be found in Supplementary online (Appendix A).

In our study, “state sequence” or “trajectory” encapsulates the dynamic changes in ADL disability states for each resident over time, capturing both progression and recovery. For instance, Sequence (I) illustrates a resident’s trajectory in our dataset. This trajectory began with state *GTBDLU* (numerical code 476), lasting 974 days. It then transitioned to state *GTBD* (code 92) for 83 days, followed by a recovery to state *B* (code 16) for 245 days, and eventually returned to state *GTBDLU* for the remainder of their stay. For a detailed explanation of the numerical coding system used to denote disability states in our trajectories, ranging from 0 (no disability) to 511 (all disabilities present), please refer to the Supplementary online (Appendix A).

The assessment records were transformed into string sequences using the TraMineR package in R^20^. As a result, a total of 148,750 state sequences were formed, representing ADL disability trajectories. These sequences span up to 5331 days, leading to the generation of a dataset of dimensions $148,750 (trajectories) \times 5,331 (days)$. Given the limitations of the SA tools available at the time of our work, handling all 148,750 sequences, some with 5331 days, posed significant computational challenges in terms of time and memory usage. To mitigate this complexity, we implemented a weighted-sequences approach to aggregate identical sequences. This method assigns weights to each unique sequence based on its frequency, thus simplifying the analysis without losing critical data insights^21^. This pre-processing step reduced the dataset by 38,794 sequences, leaving 109,956 distinct sequences for analysis.

I $$\begin{aligned} \text {Trajectory} \,\#\,\, 130202: \quad&(476,974) - (92,83) - (16,245) - (476,323) \end{aligned}$$

### Sequence analysis

SA emerged as a groundbreaking paradigm shift in social sciences, initially introduced in 1995^22^. Its versatility extends applications from prosopography^23^ to socio-economics^24^, and its strength lies in the detailed examination of complex processes. In the context of our study, SA serves as a powerful tool to dissect and understand the intricate ADL trajectories. We leverage sequence indicators, statistical measures, and visual analysis tools to unravel the sequences of ADLs, providing a nuanced understanding of these trajectories in a comprehensive manner.

#### Sequence indicators and statistical measures

Sequence indicators provide quantitative means to assess fundamental aspects of trajectories, such as their length and within-sequence diversity. These indicators are designed to capture various features of the sequences and can be grouped into four categories: basic, diversity, complexity, and (un)favorableness^25^. The “basic” indicators encompass straightforward measures, such as the proportion of states visited within a sequence. The “diversity” category can consider not only the variety of states within sequences but also their “spell duration,” which measures how long each state persists before transitioning to another within the sequence. The primary focus of the third group, “complexity” measures, is on the unpredictability of the ordering or arrangement of states. The “(un)favorableness” group of indicators quantifies the nature or quality of states within sequences, assessing them in terms of their positive or negative impact. These indicators take into account how beneficial or detrimental each state is within a sequence’s context. In our study, we examine the characteristics of the ADL trajectories by employing a variety of measures from each of these categories.

Under “basic” indicators, we measured sequence length, spell count (i.e., the number of uninterrupted periods within a sequence), number of visited states, recurrence degree (the frequency of returning to a specific state), as well as the mean and standard deviation of spell duration (i.e., the length of time a specific state persists without change). To elaborate more, a “spell” essentially represents a continuous course during which the state remains unchanged. Therefore, a spell duration refers to the length of time between each pair of transitions. Tracking the count of spells is also useful for examining transitions between states within trajectories. In the context of ADL trajectories, each spell denotes a successive period where the patient retains a particular combination of disabilities.

To evaluate the states’ variability or diversity within trajectories, we utilized longitudinal normalized entropy^25^. This concept originates from Shannon’s entropy, a popular measure to quantify the uncertainty associated with outcomes^26^. The entropy of a trajectory is measured by considering the distribution of visited states, as shown in Eq. (1), where *a* is the total number of states and $p_1, p_2,..., p_a$ are their distribution probabilities. This calculation provides insight into the level of randomness in the trajectories. Higher entropy values indicate increased variability, while lower entropy values suggest a higher degree of predictability (because of low variability) in the sequence. The normalized version of this measure is obtained by dividing it by the maximum entropy, as indicated in Eq. (2). The maximum entropy value corresponds to the entropy of the alphabet, where every state has the same number of occurrences. In this context, the “alphabet” refers to all the states observed within the sequences.

1 $$\begin{aligned} H&= -\sum _{i=1}^a p_i \log p_i \end{aligned}$$

2 $$\begin{aligned} H_{norm}&= \displaystyle \frac{H}{H_{\max }} \end{aligned}$$

Among the measures of “complexity,” we selected the turbulence measure, which focuses on the state order and the variability in spell durations^25,27,28^. Turbulence provides insights into the degree of instability or changes in the arrangement of states and the durations of spells within a trajectory^27^. It is important to distinguish turbulence from entropy; while both measures provide information about the trajectories, turbulence specifically emphasizes the ordering or sequence of states. This measure has also been improved to include 0-length spells and avoid potential undesirable behavior, as pointed out in previous research^25^. The turbulence measure, $T^*(x)$, is calculated using Eq. (3), where $\Phi (x)$ denotes the number of distinct sub-sequences within the sequence *x*^29^, and $s_d^{*2}$ represents the duration variance, including non-visited states. To elaborate further, let’s examine a sample sequence represented as $(0,4071)-(16,33)-(124,49)$. Within this sequence, considering the distinct successive states and their orders, we can identify eight different sub-sequences: an empty one ($\{\}$), individual states (0, 16, and 124), direct transitions (0-16 and 16-124), transitions through intermediate states (0–124), and the full sequence (0–16–124). Therefore, for the given sequence, $\Phi (x)$ equals 8. Also, the equations to calculate the duration variance and its maximum value ($s_{d,\max }^{*2}$) can be found in the Supplementary online (Appendix B).

3 $$\begin{aligned} T^*(x) = \log _2 \left( \Phi (x) \displaystyle \frac{s_{d,\max }^{*2}(x) + 1}{s_{d}^{*2}(x) + 1} \right) \end{aligned}$$

Furthermore, a normalized version of the turbulence measure has been developed, enhancing the comparison between trajectories. Equation (4) shows the normalized turbulence, denoted as $T^*_n$, where $T^*_{\max }$ represents the maximum value of the turbulence^25^. To compute this value, a particular sequence is formed using the alphabet (all states with a size of *a*) and the sequence’s total length (*l*). This sequence is created by repeating the alphabet [*l*/*a*] times and adding the first $l-a[l/a]$ states from the alphabet. Once this sequence is generated, its turbulence is calculated as $T^*_{\max }$. An illustrative example can be found in the Supplementary online (Appendix B).

4 $$\begin{aligned} T^*_n = \displaystyle \frac{T^* - 1}{T^*_{\max } - 1} \end{aligned}$$

Another essential aspect to consider is the inherent quality of the disability states, which can significantly differ from one another. The insecurity index is designed to comprehensively evaluate the (un)favorableness of trajectories^25^. This measure, as outlined in Eq. (5), is composed of three key components: the undesirability of states, inclination towards degradation, and instability. The insecurity index basically examines instability by using the complexity index (the third term of the equation) adjusted by the degradation index (the second term). The reason to use the degradation index is to differentiate complexity between unfavorable and desirable states. Additionally, an initial reference point for the undesirability of the states is specified by including the initial unfavorableness level (the first term).

First, we need to determine the levels of (un)favorableness among the states. We do this by considering the severity of each state, denoted by $\pi (x_a)$, which we define based on the number of disabilities within each state. In this manner, states with more disabilities are deemed more undesirable, while those with fewer disabilities are considered less undesirable. For example, states with eight disabilities (e.g., *SGTBWDLU*) have an undesirability degree of 8, while states with one disability (e.g., *G*) have an undesirability degree of 1. This approach provides an effective means to capture the relative preference of each state based on the number of functional disabilities.

The first term essentially brings the (un)favorableness of the initial spell into the equation by incorporating $\pi (x_1)$. The reason to have this value is to differentiate between complexities emerging from unfavorable states and those arising from more desirable states. The value of $\pi (x_1)$ is adjusted by multiplying by $I_{integr}(x,sp(x_1))$. $I_{integr}$, also known as integrative potential or capability^30^, is a more basic quality index that measures the tendency to transition to and remain in a positive state. Specifically, $I_{integr}(x,sp(x_1))$ quantifies the potential to integrate a spell of the first state ($sp(x_1))$). Therefore, by using the first term, the initial transition is treated as a shift from the most favorable state to the sequence’s first state ($x_1$).

The second part of the equation, the degradation index, takes into account the ratios of upward and downward state transitions based on the states’ preference order. This is achieved by subtracting the proportion of positive changes from that of negative ones, where an improvement results in a negative value. Consequently, this index produces values between − 1 and 1, where a value of − 1 indicates a sequence comprising solely upward transitions. This measure is valuable in determining whether a situation is improving or deteriorating and calculating the magnitude of change occurring over time.

Lastly, the complexity index, constituting the third term, uses the normalized within-sequence entropy adjusted for the ordering of the states (through multiplication with the proportion of transitions)^31^. This aspect reflects the unpredictability in the progression of disabilities. For more detailed information on each of these terms, please refer to the Supplementary online (Appendix B).

5 $$\begin{aligned} I_{insec}(x) = \pi (x_1) I_{integr}(x,sp(x_1)) + I_{degrad}(x) + c(x) \end{aligned}$$

#### Visual analytics

We use a variety of visualization functionalities to analyze different aspects of the trajectories^20,32,33^:

*Trajectory sampling* Usually, when handling a large number of sequences, displaying them all in a single plot can be overwhelming. To address this, we adopt an approach where the sequences are ordered based on states, and then a subset of 250 sequences are extracted at constant intervals. In other words, we arrange the sequences alphabetically based on their states. Subsequently, we group every *N*/250 sequence and visualize the first sequence of each group on the plot. This strategy offers an overview of the bulk of sequences, enhances visual clarity, and mitigates issues of over- or under-plotting. Moreover, our approach maintains computational efficiency even when dealing with a substantial number of sequences, distinguishing itself from other methodologies that tend to become computationally expensive under similar conditions. This index plot provides a comprehensive and informative visualization of the sequence structure, enabling us to discern broader patterns and trends within the ADL trajectories more effectively without the clutter of displaying all sequences. A vertical line in this index plot represents one (weighted) trajectory over time. It is worth mentioning that the weighted trajectories do not necessarily correspond to one individual path but may represent similar sequences, assigning them proportional significance based on their frequency or relevance in the dataset.

*State distribution analysis* This analysis focuses on the proportion of each disability state at different time points, offering insights into how the prevalence of each state varies over time. It helps in understanding the evolving trends of disability combinations.

*Frequency analysis* This method calculates how often each trajectory occurs within the dataset and identifies the most frequent trajectories. The visual representation of this analysis displays the relative frequency of the most observed trajectories, with wider bars indicating higher frequency. This analysis is of significant value across various applications, as it offers useful insights into the typical sequential patterns and trends present in the dataset. It is worth noting that we previously assigned weights to aggregate identical trajectories and adopted a weighted sequence analysis approach. Consequently, the most common trajectories are those with the highest weight values, which can be readily extracted and visualized without the need for re-counting trajectory occurrences. While “State Distribution Analysis” looks at the changing proportions of states over time, “Frequency Analysis” identifies and visualizes the most common overall trajectories in the dataset.

*Time spent analysis* This analysis involves computing and presenting the average time spent on each disability combination. The corresponding plot consists of vertical bars positioned from left to right in descending order. Each individual bar represents the mean duration spent in a specific state or disability combination. This graphical representation effectively highlights the long- and short-lasting states.

*Transition rate analysis* This analysis is independent of the time dimension and focuses on examining the transition rates between different states through a bubble plot. The bubbles that run diagonally indicate stability or a lack of change in the state. However, as we are primarily interested in the transitions between distinct states, we do not include the diagonal bubbles in our analysis. The size and color of the bubbles are proportional to the transition rates, providing insights into the magnitude and frequency of state changes. The vertical axis corresponds to the preceding states, while the horizontal axis corresponds to the destination states. This analysis can be valuable in understanding the disability progression and enable a straightforward tool for predicting future states based on an individual’s current state. It is essential to note that relying solely on transition counts might not ensure accurate predictions, and this analysis does not encompass the full sequential information represented by trajectories.

### Ethics declarations

Ethics approval is not required due to the data’s public accessibility.

## Results

Table 1 provides summary statistics of the basic characteristics of trajectories in our dataset. In the first column labeled “Sequence Length,” the sequence length (in days) is presented, revealing that patients were typically assessed for over two years. The next column, named “Count of Spells,” indicates the number of spells within sequences. On average, residents encountered three spells, noting that the non-consecutive spells could contain the same or distinct states. Also, the maximum number of spells recorded is 29, suggesting that some patient(s) experienced up to 28 state transitions. This high number of transitions is a noteworthy finding, warranting additional investigation to understand its clinical significance.

Moving to the third column, titled “Count of Visited States,” we observe the number of states undergone by residents. It shows that the average number of distinct states visited by patients is approximately three. Finally, the last column presents the recurrence degree, revealing that the majority of patients encountered each disability state only once. However, some patient(s) had repeated visits to specific disability combinations. A notable example is the trajectory shown in sequence (II), which has a high recurrence degree of 6, alternating between states *FGTBWDLU* (numerical code 509) and *All* (numerical code 511) multiple times. For a visual representation of this trajectory, please refer to Supplementary Fig. S1 online (Appendix C).

II $$\begin{aligned}&\text {Trajectory}\, \#\, 53209: \quad (509,941) - (511,87) - (509,259) - (511,88) - (509,81) - (511,422) - (509,83) \\&\quad - (511,161) - (509,84) - (511,259) - (509,84) - (511,251) \end{aligned}$$

The second piece of the table presents the mean and standard deviation of spell durations. The first two columns reveal that, on average, residents remained in a single state for 405 days, with a standard deviation of 213 days. This finding indicates a relatively high variance in the duration of observed spells. The last two columns consider non-visited states, highlighting the difficulty in predicting how long a state stay might extend. It is noteworthy that in the first subtable, the count of spells represents only the number of occurrences, i.e., the number of periods during which the state remains the same. In contrast, the second subtable takes into account the duration of these spells, calculating their mean and standard deviation.

**Table 1:** Basic Characteristics of ADL Trajectories.

	Subtable A					Subtable B			
	Sequence Length	Count of Spells	Count of Visited States	Recurrence Degree		MeanD^1^	Dustd ^2^	MeanD*^3^	Dustd*^4^
Min.	2.0	1.000	1.000	1.000	Min.	1.0	0.000	0.08	0.2713
1st Qu.	193.0	2.000	2.000	1.000	1st Qu.	83.0	9.708	7.68	29.7204
Median	621.0	2.000	2.000	1.000	Median	221.0	88.500	24.52	91.2587
Mean	922.3	2.959	2.565	1.113	Mean	405.1	213.278	36.00	141.1305
3rd Qu.	1384.0	3.000	3.000	1.000	3rd Qu.	503.7	301.000	54.08	205.5097
Max.	5205.0	29.000	14.000	6.000	Max.	5205.0	2455.500	208.20	1019.9675

^1^ Mean spell duration

^2^ Spell duration standard deviation

^3^ Mean spell duration, including non-visited states (0-length spells)

^4^ Spell duration standard deviation, including non-visited states (0-length spells).

**Figure 1:**
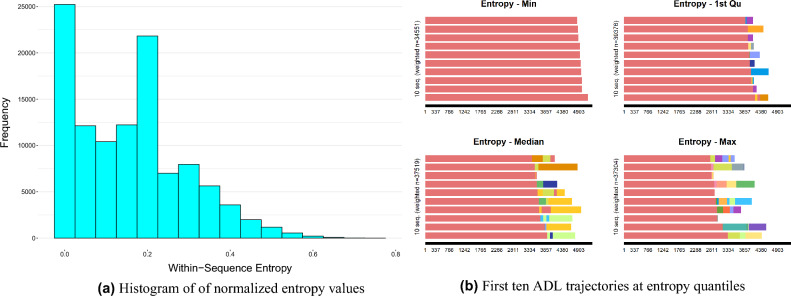
Entropy-based visual analysis of ADL trajectories (diversity analysis).

We calculated the normalized entropy for the ADL trajectories and depicted the distribution of these values using a histogram, as presented in Fig. 1a. This result reveals that most of the ADL trajectories in our dataset exhibit low entropy (with approximately 80% having entropy values less than 0.3 and 30% having values less than 0.06), indicating relatively predictable patterns. However, for the remaining 20% of trajectories, the ability to predict future disability states based only on the current state becomes more challenging and less accurate. Besides, we sought out the trajectory with the maximum entropy value, which led us to uncover numerous transitions and states within this certain trajectory. This trajectory is shown in sequence (III) and can be visually examined in Supplementary Fig. S2 online (Appendix C). As evident, trajectories of this nature present a notable challenge when predicting subsequent states based solely on preceding ones. For older adults with such trajectories, the uncertainty about future disability states would be significantly greater.

III $$\begin{aligned}&\text {Trajectory}\, \# 10162: \quad (0,337) - (16,150) - (20,720) - (116,84) - (348,238) - (92,84) - (348,77)\\&\quad - (380,84) - (476,413) - (348,161) - (92,84) - (124,245) - (126,84) - (252,77)\\&\quad - (254,84) - (252,154) - (255,167) - (511,77) - (255,84) - (254,154) - (255,147)\\&\quad - (254,114) \end{aligned}$$

To further comprehend the entropy values, it is helpful to examine the trajectories near the entropy quantiles, as displayed in Fig. 1b. This figure exhibits the first ten trajectories with their entropy values near the quantiles. The y-axis title shows the sum of weights of trajectories falling in intervals defined by each quantile, and the labels on the x-axis (1, 2, ...) represent the days. It proves that trajectories with lower entropy values have more certainty, typically containing a small number of disability states and fewer transitions between states in such cases. On the other hand, trajectories that fall within the higher quantiles of entropy carry more variability and fluctuations in their states. These trajectories display a greater diversity of disabilities and a higher frequency of transitions between various states. This observation highlights how entropy values can provide valuable insights into the predictability and variability of ADL trajectories.

We have applied a similar analytical approach to examine the complexity of trajectories using the normalized turbulence measure. Sequence (IV) shows the trajectory exhibiting the maximum turbulence value, and its visual representation can be found in Supplementary Fig. S3 online (Appendix C). This trajectory highlights multiple transitions and a more intricate progression pattern when compared to the trajectory with the highest entropy value. The individual(s) following this trajectory encountered recurrent states with varying durations during their observation period, which makes it more complex to accurately predict both future states and their corresponding spell lengths.

IV $$\begin{aligned} & {\text{Trajectory}}\;\# 86194:\quad (32,171) - (48,166) - (32,84) - (124,84) - (252,168) - (508,84) - (126,84) \\ & \quad - (508,252) - (252,84) - (126,168) - (510,84) - (508,168) - (252,84) - (508,252) \\ & \quad - (124,168) - (508,84) - (254,180) - (126,178) - (124,93) - (254,90) - (126,90) \\ & \quad - (124,180) - (126,181) - (254,672) - (510,92) - (254,188) \\ \end{aligned}$$

**Figure 2:**
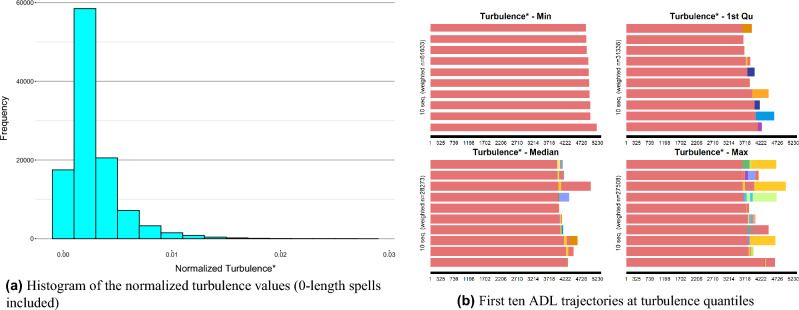
Turbulence-based visual analysis of ADL trajectories (complexity analysis).

The histogram depicted in Fig. 2a provides an overview of the distribution of normalized turbulence values. It is observable that the majority of ADL trajectories have relatively lower degrees of turbulence complexity. As mentioned earlier, turbulence assesses complexity in terms of both the ordering of states and spell durations. Furthermore, Fig. 2b reaffirms that greater turbulence values are associated with trajectories that are less ordered and predictable. An increase in turbulence diminishes the accuracy of predicting the progression pattern of disabilities, contributing to more complexity. It is notable that the overall results of the turbulence have similar behavior to those of the entropy; however, in contrast to entropy, the turbulence measure is influenced by the order of states, as evidenced by the presented trajectories and plots.

Regarding the last statistical measure, Sequence (V) illustrates the trajectory with the highest insecurity index value, and its visualization is available in Supplementary Fig. S4 online (Appendix C). Notably, this particular patient did not go through multiple transitions but instead retained the most severe state, *All* (i.e., having all disabilities, represented by numerical code 511), for an extended period.

V $${\text{Trajectory}}\;\# 17793:\;\;\;\;(0,14) - (511,426)$$

**Figure 3:**
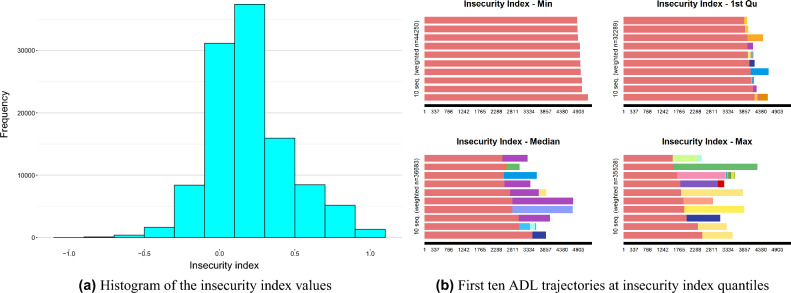
Insecurity index visual analysis of ADL trajectories (undesirableness analysis).

Finally, Fig. 3a displays the results of the insecurity index distribution, revealing that most trajectories have a low degree of unfavorableness. According to Ritschard^25^, the unbounded insecurity index might occasionally display negative values or exceed 1. However, such instances are expected to be infrequent, with most values ranging between 0 and 1. Considering the results of our analysis presented in the manuscript, we can confirm that nearly 85% of the trajectories fall within the [0,1] range. The maximum recorded value stands at 1.01, while the minimum reaches − 0.93. Furthermore, examining the plot of quantiles, Fig. 3b, confirms that trajectories with a higher level of unfavorableness often involve certain combinations, consisting of more than six ADL disabilities, and stay unchanged for prolonged durations. On the other hand, the states depicted in the median plot typically contain 3–5 disabilities or remain unchanged for shorter periods.

**Figure 4:**
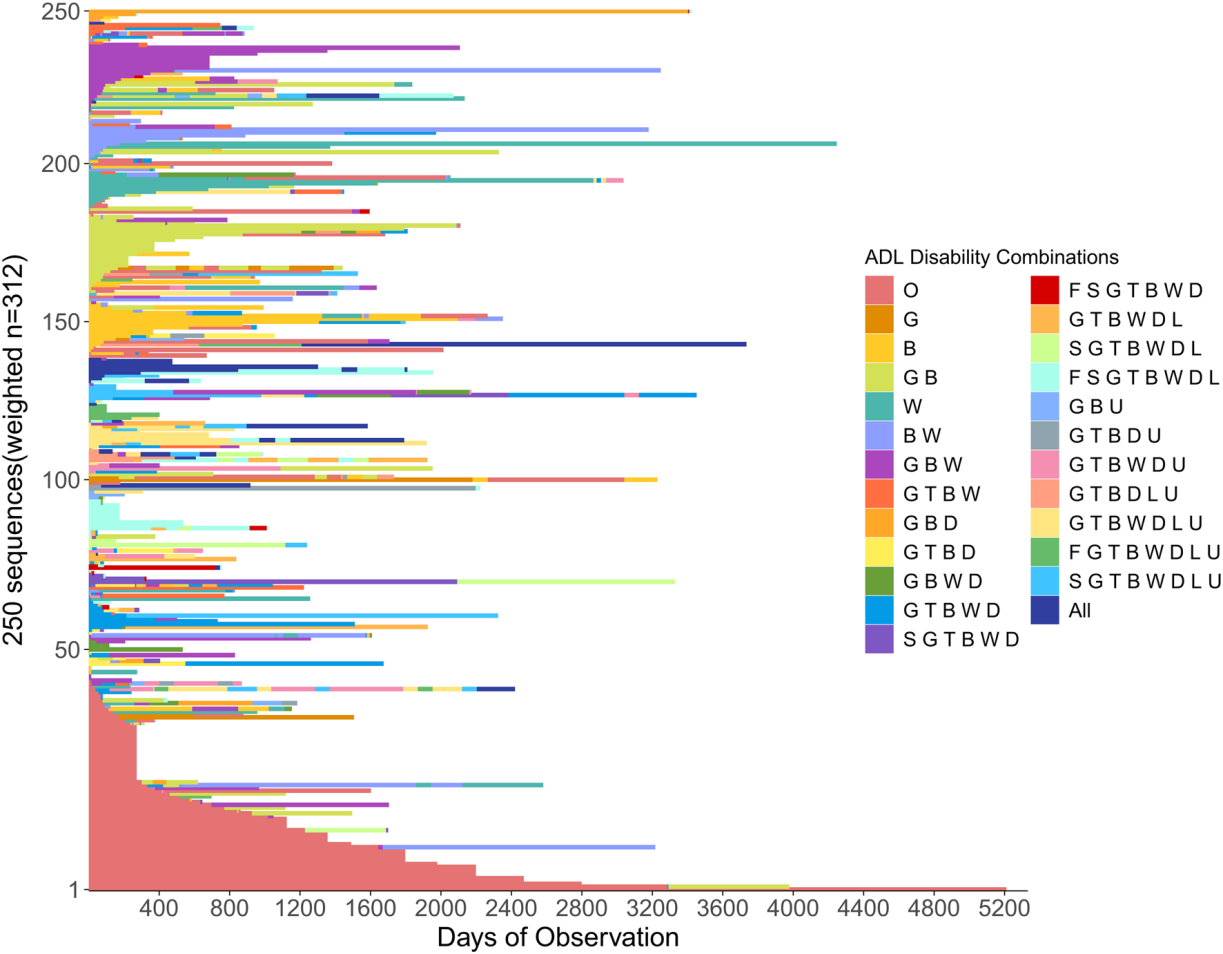
Index plot of ADL trajectories (sampled weighted sequences, sorted): showcasing time-dependent disability combinations.

Figure 4 displays the index plot of the ADL trajectories, comprising 250 weighted sequences. The time dimension, represented on the horizontal axis, and each (virtual) line along the vertical axis portrays the unique trajectory of disability combinations experienced by patients. Observing multiple colors and their varied arrangements highlights the presence of a diverse set of ADL trajectories encompassing a wide array of disability combinations. This finding suggests that older individuals may encounter many different patterns when losing their functional abilities or recovering from disabilities. Accordingly, it is unacceptable to generalize a single or a few patterns for all individuals.

**Figure 5:**
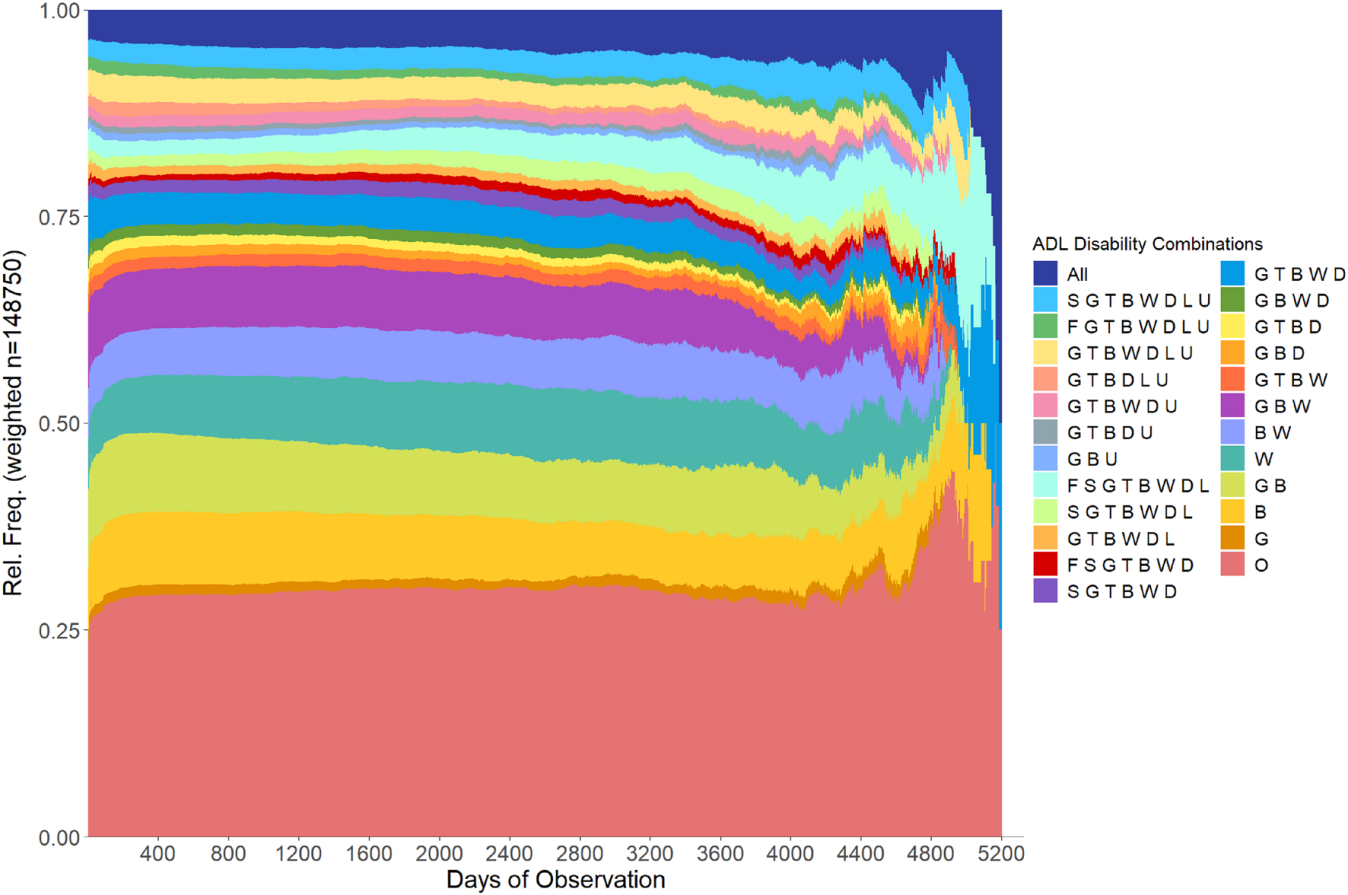
State distribution of the ADL states in our dataset.

Examination of the state distribution, as illustrated in Fig. 5, shows that a relatively stable distribution of states persists for more than 60% of the observation period. However, considerable changes become evident as we progress towards the end of the period. Remarkably, there is an increase in the proportion of the state *All* (depicted in sapphire/dark blue color at the top). This trend is also followed by the disability combinations *FSGTBWDL* (light cyan) and *GTBWD* (azure). In contrast, the proportion of some other states, such as *GBU* (Jordy blue), decreases over time. For a closer view of the legend displaying the states’ labels and colors, please refer to Supplementary Fig. S5 online (Appendix C).

**Figure 6:**
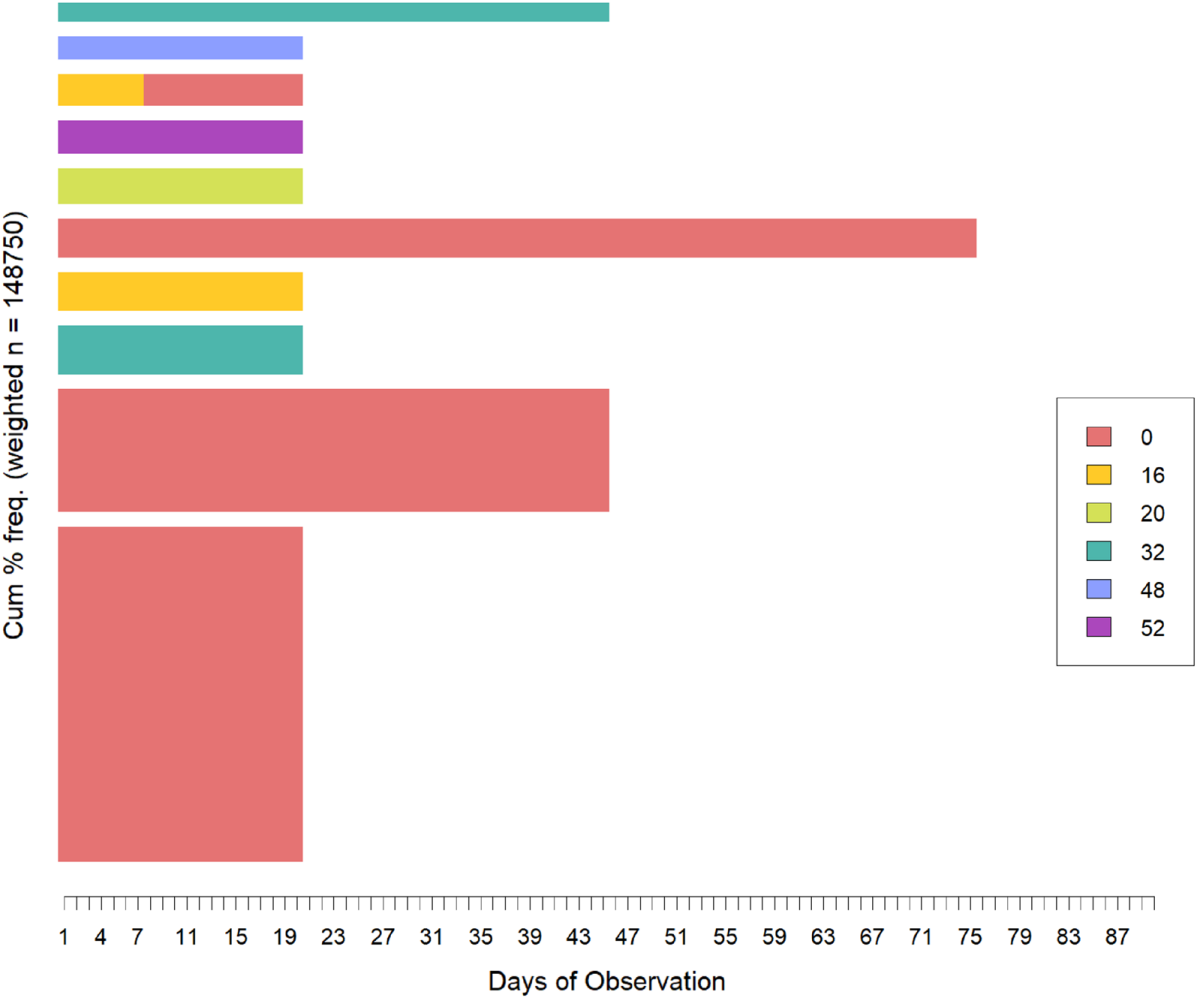
Visualization of the ten most frequent ADL disability trajectories.

Figure 6 visually presents the ten most frequent trajectories of ADL disabilities. The common trajectories typically involve a single state and unfold over relatively brief periods, with most residents experiencing no disabilities (depicted in light red) and staying in this state for a short duration, typically less than 100 days. Although this observation might appear positive, indicating that older individuals typically do not experience severe states of disability in their trajectories, it is important to consider an alternative perspective as well. This could also indicate that even apparently capable residents may have a short life expectancy in a nursing home, passing away without displaying any noticeable health status decline as a warning beforehand.

**Figure 7:**
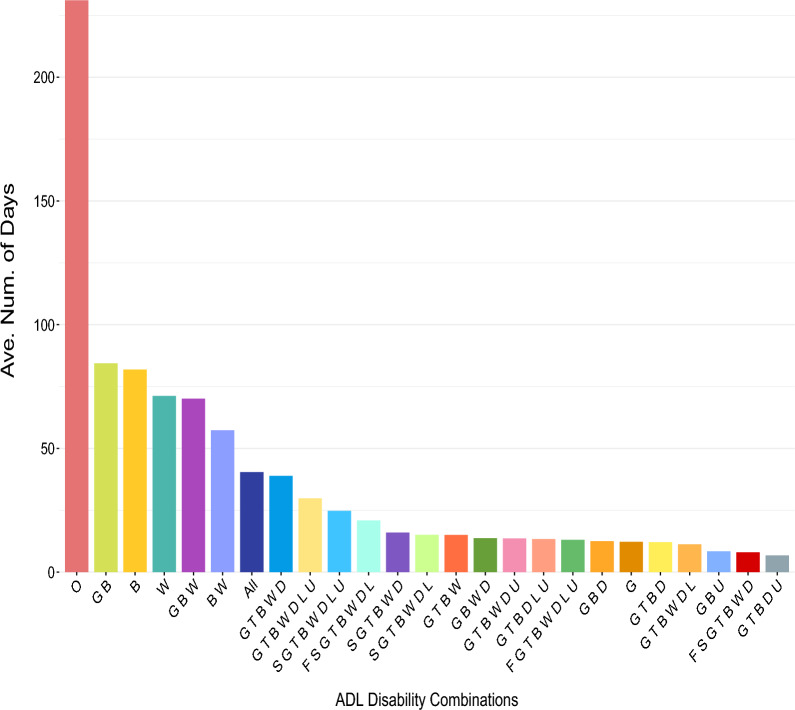
Mean time spent in each ADL state.

Figure 7 visually presents the residents’ average time in each ADL state. The results reveal that residents had the most prolonged durations in the state *O*, indicating an absence of disability. This is followed by states *GB* (i.e., Grooming and Bathing) and *B* (i.e., Bathing), which also showed relatively high durations. This finding suggests that older people might retain their disability in self-care more than in other ADL disabilities due to a decreased willingness or enthusiasm for self-care activities.

**Figure 8:**
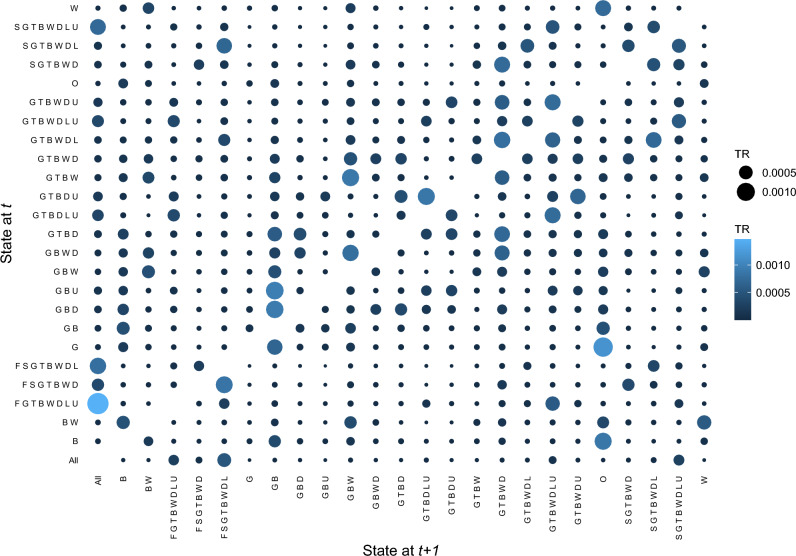
Bubble plot of transition rates between ADL states (diagonal transitions excluded).

The transition rates between various states are calculated and depicted in Fig. 8. As previously explained, this bubble plot can serve as a basic means for predicting future states. For instance, if we consider the largest and lightest blue bubble, which denotes the transition from the state *FGTBWDLU* to the state *All*, we can infer a substantial likelihood that individuals who reach the state *FGTBWDLU* will subsequently lose their ability to transfer (*S*) as well. An alternative format of this plot is provided in Supplementary Fig. S6 online (Appendix C).

## Discussion

This work analyzed trajectories of functional decline and recovery among nursing home residents. Sequence Analysis was used to examine how older adults transition among nine ADLs over time using a large dataset of disability assessments. We employed powerful sequence indicators, statistical measures, and visual analytics to investigate the ADL trajectories, aiming to enhance our understanding of the disability combinations and individuals’ pathways of functional decline and recovery. Our findings indicate that, in most cases, predicting the trajectory of ADL disabilities—how these disabilities evolve or change and their progression patterns—can be relatively manageable. This means that we can often anticipate the pattern of changes in a patient’s daily functioning abilities over time. However, accurately forecasting the duration of specific combinations of these disabilities (i.e., how long a patient will experience a particular set of ADL challenges, referred to as ADL state, before transitioning to another state) is more complex than predicting the sequence (ordering) in which they occur. Despite a thorough review of the existing literature, we found no studies explicitly addressing this complexity. This gap highlights the innovative nature of our study and its potential to significantly impact clinical practice and healthcare policy. By advancing our understanding of not only the sequence but also the duration of ADL disabilities, our findings can aid in the development of more accurate and personalized care plans. Healthcare providers can better anticipate the needs of patients over time, allowing for more efficient allocation of resources and tailored interventions.

To provide deeper insights, we tried to better understand the reasons behind the low turbulence values. To this end, we examined the distribution of the number of distinct sub-sequences ($\Phi (x)$ in Eq. (3)) and spell durations separately. Referring back to Table 1, we noted that the spell duration exhibited a relatively high level of variance. This observation prompted us to investigate the role of state arrangements reflected by $\Phi (x)$ in influencing turbulence values. Upon analyzing the distribution of $\Phi (x)$, we found that 75% of the values were less than 8, with a maximum value of 23,549,962, as shown in Table 2. This observation suggests that most of the trajectories are primarily complex in terms of spell duration but not as much in their arrangement of states. However, there are still some very complex trajectories that might need further investigation. Therefore, our analysis shows significant variability in the spell durations, which makes it challenging to estimate how long a patient will remain in a specific disability state. Finally, examining the nature of the ADL trajectories, it was found that most of them have low degrees of (un)favorableness. This suggests that patients typically experienced milder disability combinations or undesirable conditions for relatively shorter periods. However, it is important to reemphasize the size of our dataset, and the smaller proportion of trajectories with a high insecurity index still represents a considerable number. In other words, we found that there are still more than 40,000 instances of trajectories in which patients lived in very severe states and extremely unfavorable health situations.

**Table 2:** Summary of the number of distinct sub-sequences.

	$$\Phi (x)$$
Min.	2
1st Qu.	4
Median	4
Mean	1096
3rd Qu.	8
Max.	23549962

The graphs show that even when focusing on the top 25 combinations of disabilities, there is still a significant diversity in possible sequences of functional loss and recovery experienced by patients. This aligns with previous literature indicating variability in disability progression. For instance, longitudinal studies by Dunlop et al.^34^ and Jagger et al.^35^ observed distinct orders of functional disability onset in older adults but with variations in the sequence of disability occurrence. This suggests a lack of a uniform pattern in the progression of functional decline. Similarly, Gill and Kurland^36^ found no consistent sequence among their study participants, underscoring the need for a multipath analysis to understand functional loss comprehensively. Wu et al.^11^ highlighted different ADL trajectories and the importance of various predictors in understanding these trajectories, supporting the idea of variability in disability progression. Collectively, these studies corroborate our findings, indicating that the sequence in which functional disabilities emerge is highly heterogeneous. Unlike previous studies that primarily focused on the onset of single disabilities, our research contributes to this discourse by examining how combinations of disabilities emerge and evolve over time. It provides a more nuanced understanding of functional decline and recovery in patients, encompassing aspects of sequencing, timing, and duration that have not been reported in the literature.

Furthermore, disability states of *O* (no disability), *B* (bathing), *W* (walking), *GB* (grooming and bathing), *BW* (bathing and walking), and *GBW* (grooming, bathing, and walking) were the most prevalent among the residents. This implies that when residents in this setting develop disabilities, they are most likely to experience difficulties first with bathing and walking, consistent with the literature^18,35,37–39^, either individually or in combination, and potentially along with grooming. Interestingly, the state *G* (grooming disability) is a rare event in our dataset. Hence, grooming disability usually accompanies bathing or walking disabilities rather than occurring in isolation. When focusing on recoveries and employing transition rates, we were able to identify the following most common patterns:

$$\begin{array}{*{20}l} {{\text{B}} \to {\text{O}}} \hfill & {\quad {\text{GBU}} \to {\text{GB}}} \hfill & {\quad {\text{GBWD}} \to {\text{GBW}}} \hfill \\ {{\text{G}} \to {\text{O}}} \hfill & {\quad {\text{GBD}} \to {\text{GB}}} \hfill & {\quad {\text{GTBWDL}} \to {\text{GTBWD}}} \hfill \\ {{\text{W}} \to {\text{O}}} \hfill & {\quad {\text{GTBW}} \to {\text{GBW}}} \hfill & {\quad {\text{SGTBWD}} \to {\text{GTBWD}}} \hfill \\ \end{array}$$

These patterns provide insights into how individuals often recover from different disability states, such as returning to a state of no disability (*O*) after experiencing limitations in bathing (*B*), grooming (*G*), or walking (*W*), and how certain combinations of disabilities tend to be recovered over time. Speaking of recoveries, it is worth noting that within this extensive dataset of trajectories, the majority of transitions still indicated deterioration, with 45% of them representing recovery. This finding highlights that the overall probability of regaining functional abilities once lost is still relatively lower than the probability of losing them.

*Comparison of findings with prior literature* While a few studies have utilized similar datasets, they differ significantly in their approaches and objectives, highlighting the unique position of our research. Wojtusiak et al.^40^ study focused on predicting ADL changes post-hospitalization. Their approach was centered around predictive modeling using variables such as pre-hospitalization ADL status, age, and gender. The aim was to identify patterns of recovery and loss, which are significantly different from our objective of exploring the sequential nature of ADL changes. In another study, Levy et al.^18^ utilized a Bayesian Network model to describe transitions among various ADL deficits in the same VA nursing home cohort. Their study aimed at understanding the likelihood and sequence of functional decline and recovery, providing valuable benchmarks for ADL patterns. While their approach also involved analyzing ADL trajectories, it differed from ours in its focus on static network models as opposed to the dynamic temporal sequencing in our study. The research by Wojtusiak et al.^16^ shared our data source but diverged in its objective, aiming to develop an automated tool using machine learning for the prediction of ADLs. Their methodology emphasized the construction of a predictive model based on patient characteristics, differing from our study’s aim of detailing the sequence, timing, and duration of disability states. Although these studies utilized similar datasets, our study stands apart in its objective. We focus on comprehensively understanding the sequences of disability states, highlighting the timing, order, and duration of these states. This unique approach offers insights into the patterns of functional decline and recovery that extend beyond the scope of mere prediction. Our research enriches the existing literature on ADL trajectories by providing a new lens through which to view the complex progression of functional health in nursing home patients.

*Clinical and policy implications* The information gained from our sequence analysis sheds light on the characteristics and patterns of ADL trajectories, offering advantages for healthcare systems and decision-makers. For example, these findings enable us to make straightforward predictions about a patient’s trajectory based on their present state, considering transition rates and common (sub-)sequences. Additionally, this understanding equips clinicians to anticipate disability progression, tailor interventions effectively, and dynamically adapt care, ultimately resulting in improved health outcomes.

*Limitations and future research* There are areas for improvement and several promising avenues for future research and development ideas of this work. First, while removing 487 ($2^9-25$) combinations of disabilities (i.e., states) helped address complexity issues, it comes at the cost of losing information, especially regarding some particular trajectories that potentially existed in the dataset. Also, our study considered only single transitions involving one disability change at a time. Therefore, before any similar analytical work, carefully assessing the related trade-offs is imperative.

It is important to note that this study relied on a retrospective dataset where we had no control over data collection, and the assessments were conducted exclusively on veterans, leading to an imbalanced distribution of genders in the dataset, with the majority being male (97%). Besides, the veterans’ particular experiences might have affected their functional status in the long run. Therefore, future research endeavors could replicate these analyses using data collected from regular nursing homes to ensure the generalizability of the findings.

We also recommend applying our analytical approach to different subgroups of patients. However, it is noteworthy that the current dataset has limited information on the residents, containing only their age and gender data. Thus, working with an expanded dataset that includes additional personal characteristics and health-related factors could provide deeper insights and enable personalized analyses. By incorporating more features, such as demographic information, medical history, and comorbidities, we can segment the residents into subgroups and perform a comparative analysis of their trajectories of functional decline and recovery. This would enhance the comprehensiveness and depth of our findings.

Moreover, we observed a relatively consistent pattern in the distribution of states throughout the time, with only minor changes towards the end of the observation period. This could be attributed to the small number of residents who stayed for a prolonged length of time. Specifically, we found that merely 2% of the patients remained for more than 60% of the observation period (equivalent to over 8.5 years). So, we conclude that the large number of cases might obscure the variations in the state distribution, creating the impression of stability over time. Therefore, we recommend studying smaller groups of trajectories using techniques like clustering to delve deeper into the actual temporal patterns of disability states.

Although we used different methods to study ADL trajectories, we suggest examining further analyses like Principal Component Analysis (PCA) to reveal more hidden patterns. This approach can help uncover valuable insights and patterns that might not be immediately apparent through other means.

Lastly, in future research, we intend to explore ADL trajectory clustering to enrich our analytics further and enhance our understanding of functional decline and recovery trajectories. This could have significant implications for advancing clinical decision-making, improving patient outcomes, developing effective predictive models, providing patients with more personalized interventions, and ultimately elevating the overall quality of healthcare.

## Conclusions

This study provides a comprehensive analysis of ADL trajectories, offering insights into the patterns of functional decline and recovery among individuals with varying disability combinations. By employing a SA approach, we have shed light on the diversity, complexity, and nature of ADL trajectories. Our findings have also revealed the most common patterns of functional loss and recovery and highlighted the prevalence of certain states and transitions. This knowledge can significantly benefit healthcare decision-making, empowering clinicians to anticipate disease progression, tailor interventions effectively, and improve the overall quality of care.

### Supplementary Information

Supplementary Information.

## Data Availability

The dataset supporting this study is accessible through the dedicated GitHub repository created for this research (https://github.com/zargousa/ADL_assessments). To access this item, please use the password *ADL2023*.
