# Supplementary material for: Exploring trajectories of functional decline and recovery among older adults: a data-driven approach
Source: Sci Rep. 2024 Mar 15;14:6340. doi: 10.1038/s41598-024-56606-0 (PMC10943109; doi:10.1038/s41598-024-56606-0)
Supplement: Supplementary file 1 — Supplementary Information. [file 41598_2024_56606_MOESM1_ESM.pdf]

Supplementary Information

The supplementary section complements and expands upon the information presented in the main sections of the paper, titled "Exploring Trajectories of Functional Decline and Recovery among Older Adults: A Data-Driven Approach". This enhances the comprehensiveness of the research findings and aids in understanding the visual and statistical data presented in the main body of the paper.

Appendix A-Data

The data for this study were sourced from the Corporate Data Warehouse of the Department of Veterans Affairs and analyzed using the VA's Computing Infrastructure. The original data were derived from two primary sources: (1) patient records obtained from the VA's Electronic Medical Record System, and (2) assessments from the Minimum Data Set (MDS) for VA nursing homes across the nation. The MDS is a uniform and compulsory clinical assessment process for residents in nursing homes certified by Medicare and Medicaid in the United States. Established in accordance with the Omnibus Budget Reconciliation Act of 1987 (OBRA '87), Public Law (P.L.) No. 100–203, title IC, subtitle C, 101 Stat 1330, and introduced in 1991 with routine updates, the MDS facilitates comprehensive evaluations across various areas: (1) cognitive function, (2) communication and hearing capabilities, (3) vision, (4) physical abilities, (5) continence, (6) psycho-social health, (7) mood and behavior, (8) patterns in activity engagement, (9) medical diagnoses, (10) additional health conditions (such as accidents and pain), (11) nutritional and oral health, (12) dental status, (13) skin health, (14) medication usage, (15) treatments and medical procedures, and (16) activity patterns. The Veterans Health Administration employs the MDS in its Community Living Center program to align with the standards of nursing homes in the U.S. The data are collected by multidisciplinary professional staff within each facility and electronically sent to a central database for use in quality assessment and resource distribution. The reliability of MDS data is high, with intraclass correlation coefficients typically above 0.7. Per VA policy, assessments should be conducted within 14 days of admission, at least quarterly, or following significant events like hospitalization or noticeable changes in a resident's condition. However, the frequency of these assessments can vary from the policy recommendation. For more detailed information regarding the data sources, readers are encouraged to refer to the cited papers[1–3].

In the dataset, one separate column is dedicated to each ADL, with 1 denoting disability and 0 indicating its absence. We derived the disability states by employing binary coding. As a result, the disability states are represented through numbers ranging from 0 to 511, with 0 representing the "no disability" state and 511 representing "all disabilities". As an example, a patient incapable of feeding, transferring, and toileting (*FST*) would be represented by a state code of 11. It is important to note that the order of disabilities within a state holds no significance; for instance, *FST*, *FTS*, and *STF* all denote the same disability state. Furthermore, it's important to emphasize that this study specifically focuses on the top 25 most frequent states, as detailed in Table S1.

Table S1. 25 most prevalent ADL states

State	Name	Severity	State	Name	Severity
0	O	0	127	F S G T B W D	7
4	G	1	252	G T B W D L	6
16	B	1	254	S G T B W D L	7
20	G B	2	255	F S G T B W D L	8
32	W	1	276	G B U	3
48	B W	2	348	G T B D U	5
52	G B W	3	380	G T B W D U	6
60	G T B W	4	476	G T B D L U	6
84	G B D	3	508	G T B W D L U	7
92	G T B D	4	509	F G T B W D L U	8
116	G B W D	4	510	S G T B W D L U	8
124	G T B W D	5	511	All	9
126	S G T B W D	6			

Appendix B-Terms & Definitions

Table S2 presents a glossary containing general terms and their corresponding definitions utilized throughout the paper. Furthermore, Table S3 provides definitions helpful in understanding different mathematical terms and equations. For an in-depth description, please refer to the work by Ritschard (2021)[4]. Equations (S1) and (S2) show how to calculate the variance of spell duration and its maximum value, respectively. Additionally, Equations (S4), (S5) and (S6) present the formulas for the integrative potential, the degradation index, and the complexity index, which are pivotal components in establishing the insecurity index.

Table S2. Summary of terms and definitions

Term	Description
ADL Disability State	Specific combinations of ADL disabilities.
State Sequence or Trajectory	Chronological sequence of varying ADL disability states for an individual over time.
Weighted Trajectories	Sequences with varying degrees of significance, determined by their frequency within a dataset.
Sequence length	Duration, measured in days (or any chosen time units), of an individual's trajectory.
Spell	A continuous period during which a specific state remains unchanged.
Spells Count	Number of uninterrupted periods within an individual's trajectory.
Visited States Count	Number of unique ADL states encountered within an individual's trajectory.
Recurrence degree	Frequency of revisiting specific states within an individual's trajectory.
Spell Duration	Length of time an individual remains in a specific disability state within a trajectory or sequence.
0-Length Spells	Transitions between disability states without measurable duration, incorporating non-visited states into sequence measures.
Entropy	Uncertainty and variability of states within sequences.
Turbulence	Instability and unpredictability in state ordering or arrangement as well as spell durations within a trajectory.
State Distribution	Proportion of disability states at different time points within a dataset.
(Un)Favorableness or (Un)Desirableness	The positive or negative nature or quality of states within sequences.

$$s_d^{*2} = \frac{1}{l_d + n_{nv}} \left(\sum_{i=1}^{l_d} \left(d_i - \bar{d}^*\right)^2 + \sum_{i=1}^{n_{nv}} \bar{d}^{*2} \right) \tag{S1}$$

$$s_{d,\max}^{*2} = \frac{(l_d - 1)\left(1 - \bar{d}_{\max}^*\right)^2 + \max_{nv} \bar{d}_{\max}^{*2}}{l_d + \max_{nv}} \tag{S2}$$

where $\bar{d}_{\max}^*$ is the adjusted mean duration and calculated using (S3).

$$\bar{d}_{\max}^* = \frac{\bar{d}^* \left(l_d + n_{nv}\right)}{l_d + \max_{nv}} \tag{S3}$$

$$I_{integr}(x) = \frac{\sum_{i=1}^{l} is.pos(x_i) i^w}{\sum_{i=1}^{l} i^w} \tag{S4}$$

where $is.pos(x_i)$ gets a value of 1 when the x_i is a positive state and 0 otherwise. Also, w serves as a recency weighting factor that gets higher values for more recent states.

$$I_{degrad}(x) = r^-(x) - r^+(x) \tag{S5}$$

Table S3. Summary of statistical terms and mathematical notations

Term	Definition
a	Alphabet size, i.e., the total number of states
p_i	Distribution probability of state i
H_{norm}	Longitudinal normalized entropy
$\Phi(x)$	Number of distinct sub-sequences within the sequence x
s_d^*	Spell duration standard deviation, non-visited states included
$s_{d,\max}^{*2}$	Maximum variance of spell duration, non-visited states included
l	Sequence length
l_d	Number of spells within the sequence
d_i	Duration of the ith spell
$\bar{d}^*$	Mean duration of spells, non-visited states included
n_{nv}	Number of non-visited states
$\max_{nv}$	Maximum number of non-visited states (equals $a-1$ when $l_d = 1$ and $a-2$ O.W.)
$\bar{d}_{\max}^*$	Adjusted mean duration
$T_{\max}^*$	Maximum turbulence
x_i	ith element (state) in the sequence x
$\pi(x_i)$	(Un)favorableness degree of state x_i
$I_{insec}(x)$	Insecurity index of sequence x
$I_{integr}(x)$	Integrative potential/capability of sequence x
$sp(s)$	A spell with the state s
$I_{degrad}(x)$	Degradation index of sequence x
$r^{-(+)}$	Proportion of negative(positive) transitions
$c(x)$	Complexity index of sequence x
tp	Proportion of transitions

where r^- denotes the proportion of negative changes, and r^+ is that of the upward transitions.

$$c(x) = \sqrt{tp(x)H_{norm}(x)} \tag{S6}$$

As an illustrative example of generating the particular sequence to compute $T_{\max}^*$, let's consider a sequence of size 10 with an alphabet comprising three states (0, 4, 16). This sequence is created by repeating the alphabet $[l/a] = [10/3] = 3$ times, resulting in (0-4-16-0-4-16-0-4-16). Then, the first $l - a[l/a] = 10 - 3 \times 3 = 1$ states from the alphabet (0) are added to the sequence. Subsequently, the turbulence (T^*) is calculated for the derived sequence (0-4-16-0-4-16-0-4-16-0) and considered as the maximum turbulence value for the initial sequence.

Appendix C-Figures

Figures S1, S2, S3, and S4 showcase trajectories with the highest values of recurrence degree, entropy, turbulence, and insecurity index, respectively. Moreover, Fig. S5 offers a legend illustrating the various states and their corresponding colors used throughout the main sections, serving as a reference for interpreting the graphical data. Finally, Fig S6 presents the transition plot in another format, with percentage values displayed in each cell.

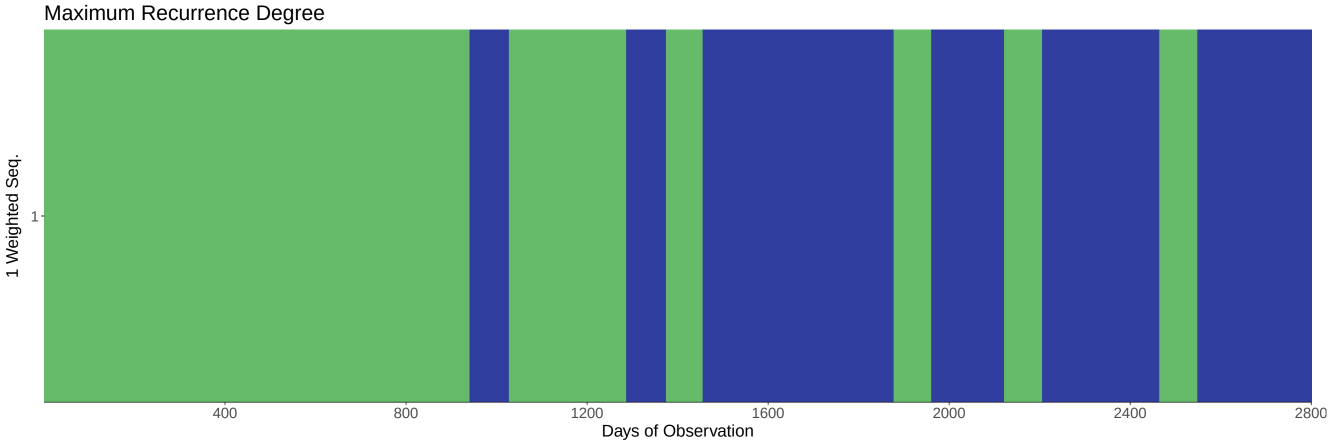

Figure S1. Trajectory with maximum recurrence degree

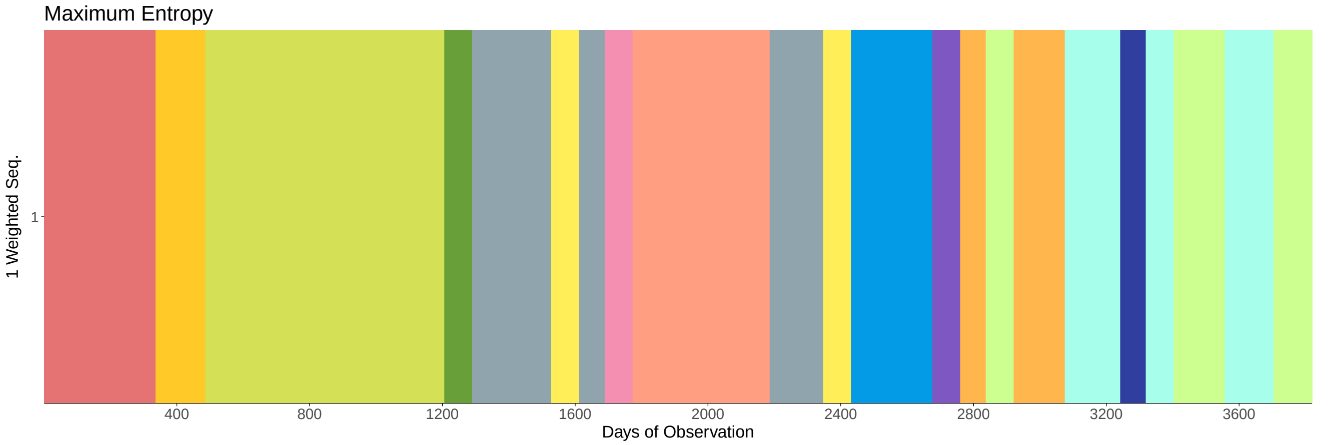

Figure S2. Trajectory with maximum entropy

References

1. Levy, C. R. *et al.* Sequence of Functional Loss and Recovery in Nursing Homes. *The Gerontol.* **56**, 52–61, DOI: 10.1093/geront/gnv099 (2016).
2. Wojtusiak, J., Levy, C. R., Williams, A. E. & Alemi, F. Predicting Functional Decline and Recovery for Residents in Veterans Affairs Nursing Homes. *The Gerontol.* **56**, 42–51, DOI: 10.1093/geront/gnv065 (2016).
3. Wojtusiak, J., Asadzadehzanjani, N., Levy, C., Alemi, F. & Williams, A. E. Computational Barthel Index: an automated tool for assessing and predicting activities of daily living among nursing home patients. *BMC Med. Inform. Decis. Mak.* **21**, 17, DOI: 10.1186/s12911-020-01368-8 (2021).
4. Ritschard, G. Measuring the Nature of Individual Sequences. *Sociol Methods Res* 00491241211036156, DOI: 10.1177/00491241211036156 (2021).

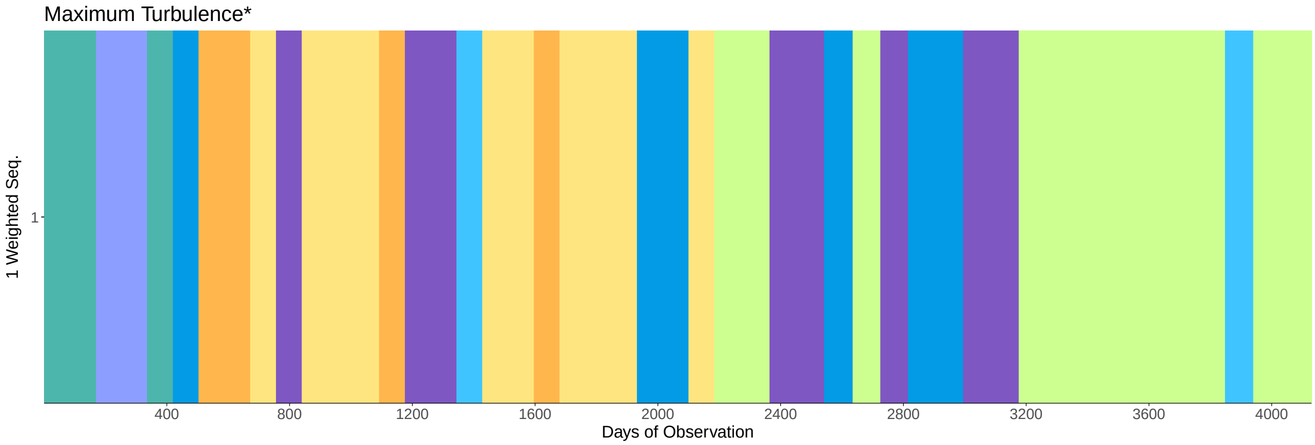

Figure S3. Trajectory with maximum turbulence

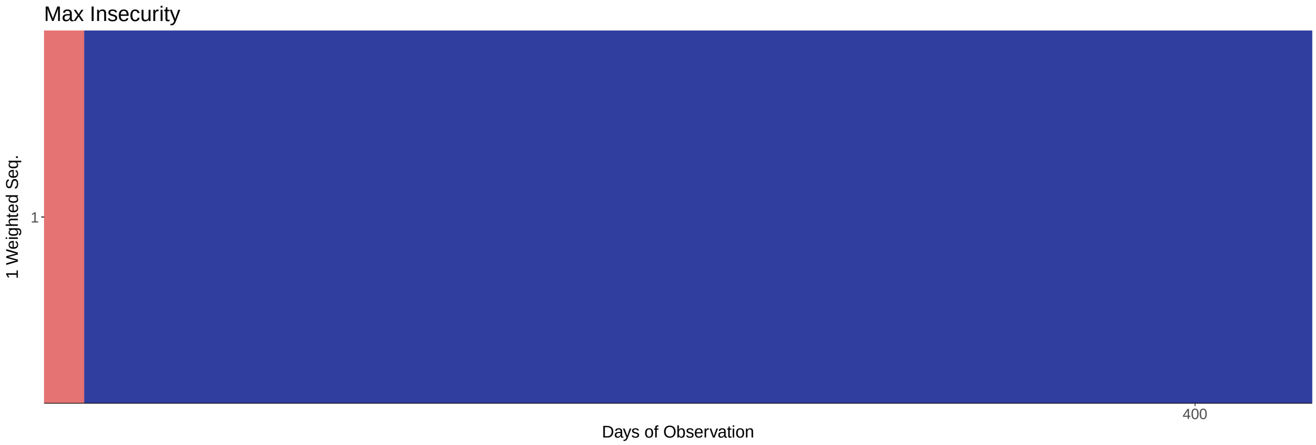

Figure S4. Trajectory with maximum insecurity index

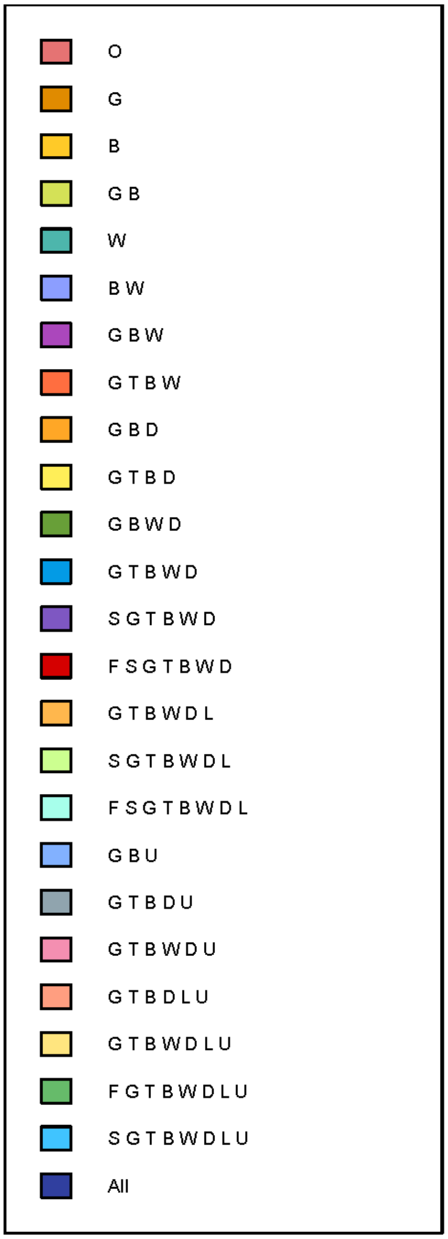

Figure S5. Legend for the states (disability combinations)

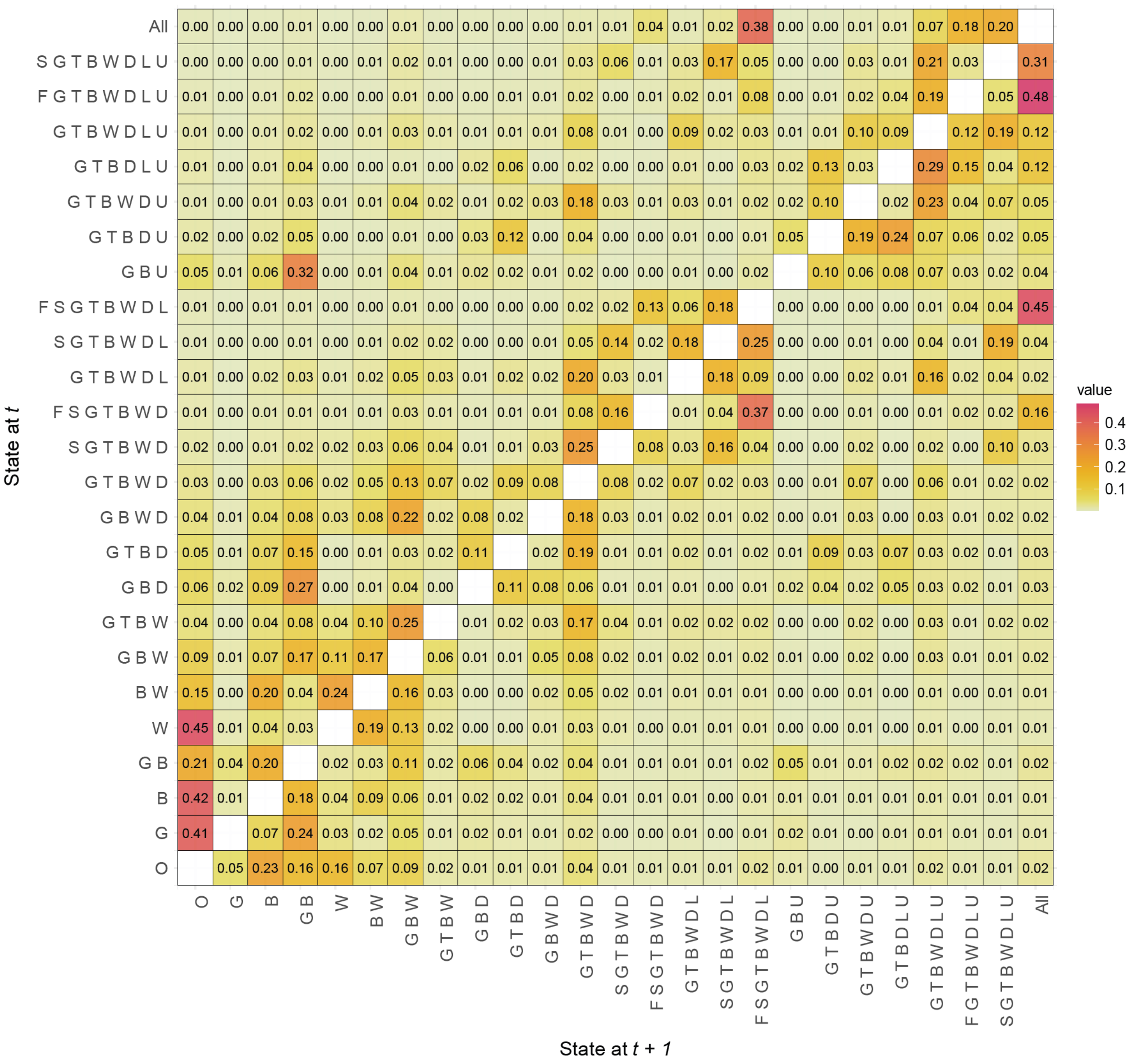

Figure S6. Transition rates between different states
